# Personalized Telehealth in the Future: A Global Research Agenda

**DOI:** 10.2196/jmir.5257

**Published:** 2016-03-01

**Authors:** Birthe Dinesen, Brandie Nonnecke, David Lindeman, Egon Toft, Kristian Kidholm, Kamal Jethwani, Heather M Young, Helle Spindler, Claus Ugilt Oestergaard, Jeffrey A Southard, Mario Gutierrez, Nick Anderson, Nancy M Albert, Jay J Han, Thomas Nesbitt

**Affiliations:** ^1^ Laboratory of Assistive Technologies - Telehealth & Telerehabilitation, SMI Department of Health Science and Technology Aalborg University Aalborg Denmark; ^2^ Center for Information Technology Research in the Interest of Society (CITRIS) UC Berkeley Berkeley, CA United States; ^3^ Faculty of Medicine Qatar University Qatar Qatar; ^4^ Center for Innovative Medical Technologies (CIMT) Odense University Hospital University of Southern Denmark Odense Denmark; ^5^ Connected Health Innovation Partners HealthCare Harvard Medical School Boston, MA United States; ^6^ Betty Irene Moore Nursing School Davis Health System University of California Sacramento, CA United States; ^7^ Department of Psychology and Behavioral Sciences Aarhus University Aarhus Denmark; ^8^ Citizens & Labor Market Esbjerg Municipality Esbjerg Denmark; ^9^ Division of Cardiovascular Medicine University of California Davis Medical Center Sacramento, CA United States; ^10^ Center for Connected Health Policy Sacramento, CA United States; ^11^ Department of Public Health Sciences School of Medicine University of California Sacramento, CA United States; ^12^ Cleveland Clinic Health System Cleveland, OH United States; ^13^ Center for Health and Technology Davis Health System UC Berkeley Sacramento, CA United States

**Keywords:** telehealth, research, individualized medicine, telemonitoring, prevention, mobile phone

## Abstract

As telehealth plays an even greater role in global health care delivery, it will be increasingly important to develop a strong evidence base of successful, innovative telehealth solutions that can lead to scalable and sustainable telehealth programs. This paper has two aims: (1) to describe the challenges of promoting telehealth implementation to advance adoption and (2) to present a global research agenda for personalized telehealth within chronic disease management. Using evidence from the United States and the European Union, this paper provides a global overview of the current state of telehealth services and benefits, presents fundamental principles that must be addressed to advance the status quo, and provides a framework for current and future research initiatives within telehealth for personalized care, treatment, and prevention. A broad, multinational research agenda can provide a uniform framework for identifying and rapidly replicating best practices, while concurrently fostering global collaboration in the development and rigorous testing of new and emerging telehealth technologies. In this paper, the members of the Transatlantic Telehealth Research Network offer a 12-point research agenda for future telehealth applications within chronic disease management.

##  Introduction

Telecommunication technologies have been used to bring health care expertise to the point of care since the 19th century. In 1878, *The Lancet* reported on the use of the telephone to reduce unnecessary physician visits and, in 1910, a tele-stethoscope had already been described [1]. During the mid-20th century, NASA used remote monitoring systems to measure astronauts’ physiological functions. The Space Technology Applied to Rural Papago Advanced Health Care project further developed this field with the Papago Indians in the southwestern United States [2]. However, the greatest strides in the use of remote monitoring technologies for telehealth have occurred over the last 10 years, with a growing evidence base showing their effectiveness in the management of chronic disease [3-5].

The importance of telehealth as a major vehicle for delivering timely care over distance has become increasingly relevant as the world’s health care needs have become overwhelmed by a significant increase in the global level of chronic disease. Chronic disease now exceeds communicable disease as the leading cause of death worldwide. In the United States, more than 70% of deaths are associated with chronic diseases and approximately 75% of annual health care expenses are used on persons with chronic conditions [6,7], a problem that is increasing as the prevalence of chronic diseases grows with aging. In the European Union, it is estimated that chronic illness is a factor in 87% of all deaths [8].

Some telehealth models of care have shown clear benefits for patients with chronic disease that incorporate patients and family members into the care team [9], whereas others have not been able to demonstrate significant improvements [10]. These models of care, which frequently involve remote patient monitoring (RPM), show promise in getting and maintaining patients to achieve their health care goal and, in some cases, lowering the incidence of avoidable hospitalizations and rehospitalizations for patients with chronic conditions [3,10-12]. In the United States and the European Union, telehealth technologies have also been shown to be effective in small-scale studies of patients with chronic diseases; nevertheless, adoption of telehealth solutions remains limited [5,13,14]. There are several obstacles to achieving widespread adoption of telehealth: acceptance of this technology by patients and clinicians, economically sustainable reimbursement systems, interoperability between electronic patient record systems, and technological capacity to accommodate bandwidth-heavy telehealth programs in smaller hospitals, clinics, and in the home.

### Purpose

This paper was prepared by an international team of telehealth providers, clinicians, researchers, and policy analysts assembled through the Transatlantic Telehealth Research Network (TTRN). The mission of the TTRN has been to design a future agenda for telehealth innovation and research. The TTRN was established in 2012 to link major institutions in the United States, Denmark, and the European Union. The aim of the TTRN is to develop cutting-edge telehealth research and innovation within telehealth that can be translated into practice and rapidly scaled up. Using an interdisciplinary approach, TTRN members focus on problem-based research and on developing new diagnostic, preventive care, and treatment methods and technologies for patients through telehealth systems.

This paper has two aims:

1. To describe the challenges of promoting telehealth implementation and advancing adoption.
2. To present a global research agenda for personalized telehealth within chronic disease management.

Using evidence from the United States and the European Union, this paper provides a global overview of the current state of telehealth services and benefits, presents fundamental principles that must be addressed to advance the status quo, and provides a framework for current and future research initiatives within telehealth for personalized care and treatment.

## Telehealth Today: Benefit and Biases

In 2008, a meta-analysis of home monitoring studies globally found it to be a cost-effective alternative in 21 of 23 studies, the majority of which focused on chronic disease care [15]. The main benefits derived from RPM programs were decreased hospital utilization, improved patient compliance with treatment plans, improved patient satisfaction with health services, and improved quality of life. Multiple studies have found savings associated with the application of telehealth for home monitoring when applied to heart failure patients. A set of recent studies that compared telehealth to traditional outpatient care recorded savings estimates ranging from 17% to 75% [16-22]. Similarly, a recent comprehensive review of telehealth studies among patients with chronic obstructive pulmonary disease (COPD), heart failure, and stroke concluded that there were reductions in hospital admissions/readmissions, length of hospital stay, emergency department visits, and often a decrease in mortality [23].

Although benefits of telehealth were cited in these studies, Wootton [5] identified a publication bias in telehealth studies of chronic disease management, with 108 of 110 articles reporting positive effects. Telehealth studies were characterized by a very short duration, averaging 6 months, and there were few studies of cost-effectiveness. Wootton concluded that the evidence base of telehealth and chronic disease management was both contradictory and weak [5].

A review of the cost-effectiveness of telehealth [24] concludes that economic tools for evaluation are being increasingly used, but better reporting of methodologies and findings is required in future research. The review also concluded that there was no convincing evidence to show that telehealth was more cost-effective than conventional health care [5].

In the United Kingdom, a large telehealth project, the Whole System Demonstrator (WSD) project, was carried out with 3230 patients between 2008 and 2009, including those with heart, lung, and diabetes diseases using a cluster randomized design. During a 12-month observation period, researchers found reductions in mortality and hospital admissions [25]. The effects on health-related quality of life were shown not to be significant [26]. An economic analysis of the WSD concluded that telehealth was not cost-effective when used as an add-on to standard care and treatment for patients [27]. In addition, a longitudinal case study of the organizational effects of the WSD showed that the randomized research design impeded organizational learning among the trial sites and that the full organizational benefit of WSD was not achieved [28]. In the WSD, qualitative interviews were carried out with those patients who declined to participate in the study. Among this group, 36.7% chose not to participate in the study following a home visit in which they had been informed about the study. Through interviews with this group, their primary concerns were shown to be threats to identity, independence, and self-care; requirements of technical competence and ability to operate equipment; and experiences of disruption of health and social care services [29]. The implication is that there is a bias in telehealth studies and the samples studied tend to consist of people who were already positively disposed to telehealth measures.

In a systematic review focusing on patients’ acceptance of telehealth technologies, the authors concluded that focusing on patient factors alone was not sufficient for understanding the degree of patients’ interest (or lack of interest) in using telehealth technologies [30]. Future research is needed to identify additional factors that promote telehealth acceptance, such as human-technology interaction, organization of the health care system, and social factors. We do not claim that telehealth should be held to a higher evaluation standard than what has been applied to traditional modes of care. However, investigating additional features that affect the success of telehealth utilization will enable more informed development of future telehealth implementations.

### Telehealth Challenges

Health care financing models exist in many industrialized nations. In the United States in 2011, a national quality strategy for establishing aims and priorities for quality improvement, known as “the Triple Aims” [31], included improving the overall quality of health care, better population and community health outcomes, and reducing the overall cost of care. In both the United States and the European Union, it is clearly anticipated that technology will play a pivotal role in achieving these goals.

Nevertheless, even with governmental support and a growing evidence base demonstrating the benefit of health care financing models of care that included telehealth, there remain many challenges facing the establishment and sustainability of effective telehealth programs. Financial sustainability of telehealth models of care has been one of the main challenges, particularly in the United States, where—despite the intent of the Affordable Care Act—reimbursement has been and continues to focus more on paying for care processes that occur within health care facilities rather than care processes that affect patient outcomes. Although reimbursement for telehealth increased in the United States, payers such as Medicare do not recognize the home as a reimbursable originating site of care. In settings with capitated reimbursement, such as the US Veterans Administration, the use of telehealth solutions (RPM and home-based chronic disease management) has had remarkable success [3]. Among 17,025 adults, researchers found a 25% reduction in hospital length of stay, a 19% reduction in hospital admissions, and a mean satisfaction score rating of 86%, all at a cost of US $1600 per patient per annum [3].

There are clearly additional challenges beyond reimbursement to bringing telehealth models to scale across different types of health systems and independent practitioners in the United States and the European Union. Most successful telehealth models require an extensive care team, including disease management nurses and other personnel. Independent practitioners may not be able to employ care teams and would potentially need to rely on an intensive service model, such as visiting nurses for home health care. Effective implementation of telehealth often requires receiving and processing data from various devices, which need to be analyzed and translated into actionable clinical information for physicians and other health care providers.

To put it simply, if data from RPM and other telehealth technologies are to be used for clinical decision making, the clinician must be assured that physiological and activity data are accurate. Initiatives such as the Personal Connected Health Alliance are intended to assure that this occurs. As the use of RPM and telehealth becomes more standardized and ubiquitous, and as health data are collected and stored in standard formats, there are considerable opportunities to apply predictive analytics. Ideally, clinicians should have easily interpretable dashboards identifying the daily health statuses for all their chronic disease patients. Some organizations have already begun developing and using chronic disease dashboards for conditions such as diabetes [32]. There are also opportunities to adapt off-the-shelf technologies, such as mobile phones and gaming systems, to serve as tools in remote chronic disease management. Ultimately, in order for technology-enabled chronic disease models to be adopted on a large scale, more research is needed to determine health care professionals’ and patients’ preferences for technologies and care models and methods to assure accurate data.

As new technology solutions, such as sensors, mobile devices, and self-tracking technologies, become more prevalent, organizations will increase use and reimbursement of technology-driven health care services. As technology-driven health care services grow, this will require development of efficient business models and cases for telehealth stakeholders.

### Policy Challenges Within Telehealth

Telehealth policies among US states, US federal agencies, and in EU countries are outdated and woefully inadequate to support widespread telehealth adoption and growth. In 2015, the California-based Center for Connected Health Policy (CCHP) undertook a comprehensive analysis of the laws, regulations, and related administrative policies of the 50 states in the United States as well as the federal US policy [33]. Given that each state can define its own policies for its Medicaid Program, a wide range of definitions and reimbursement policies for telehealth care were found, with no two states being alike. Based on the CCHP review, there are several critical policy issues that require attention: (1) defining telehealth care, (2) setting reimbursement policies, (3) licensing and jurisdictional issues, and (4) cost-benefit analysis of telehealth systems. It is hoped that refinements in these state policy initiatives will enable the achievement of the following three goals:

1. Creation of parity for telehealth with other modes of health care delivery;
2. Active promotion of telehealth as a tool to advance stakeholder goals regarding health status, equity of access, greater efficiency in care delivery, and health systems improvements; and
3. Creation of opportunities and flexibility for telehealth to be used in new models of care and systems improvements.

The US federal policy governing the use of telehealth in the Medicare program is also very limited. Reimbursement is limited to certain billing codes and only for live video care. In addition, these services are limited only to beneficiaries who reside in strictly defined rural communities.

An analysis of the eHealth policy initiatives within 27 EU Member States influenced the development of an eHealth roadmap that reflects national, regional, and local conditions that go beyond technical imperatives to include personalized telehealth solutions [34]. Commonalities among US and EU telehealth policies exist at the national and regional levels resulting in generally uniform policy solutions; however, at the local level there tends to be a lack of personalized telehealth solutions.

## Definitions and Nomenclature

In a 2014 study [35], 26 US federal agencies were surveyed and seven distinctly unique working definitions of “telehealth” were identified. The study concluded that a common nomenclature for defining telehealth may benefit efforts to advance the use of this technology so that it can address the changing nature of health care and the emerging demands for services as a result of health care reforms [35]. EU telehealth definitions and applications reveal similarly wide variation of terms and restrictions in use [36]. Internationally, there is neither a common understanding of the various forms of technology-enabled health care nor precision in the description of the wide range of health-related activities and services that are covered via telehealth.

## Reimbursement

Within the United States and the European Union, reimbursement of telehealth-delivered care and specific reimbursement requirements remain a major challenge. A legal definition of telehealth may relate directly to services that will be reimbursed by public and private payers and the conditions for this reimbursement. It would be beneficial if reimbursement policies, in addition to including live video, were consistent within and between countries and included asynchronous “store-and-forward” and remote monitoring. Policies should also be sufficiently flexible to create parity and should not be restricted by artificial barriers such as geographic limitations (as is the case with Medicare in the United States). Technology-enabled health care should be seen as a virtual modality, not a distinctly separate service requiring unique billing codes. The impact of telehealth-enabled health care will become more of a reality in the United States as health systems shift from fee-for-service to more value-based capitated systems under health care reform. In the European Union and other countries with nationalized health care, opportunities for incorporating telehealth practices in innovative reimbursement schemes are being advanced within different health care systems.

## Licensing and Jurisdictional Issues

The scaling of telehealth, particularly in the United States, has been limited by professional licensing issues and competition among professionals. The ability to provide high-quality virtual synchronous and asynchronous health care and patient monitoring has created unprecedented opportunities for dramatically expanding access to quality care for the underserved and simultaneously increasing the efficiency and lowering the costs of care [37]. Services can now be effectively provided across the street and around the planet as long as there is access to high-speed Internet. However, the definition and interpretation of the practice of medicine in the United States is determined at the state level and is defined by each state medical board, thus resulting in significant limitations in geographic and population scaling. Similarly, health care systems vary from country to country. There is no question that the rapid growth of technology-enabled health care will create increased pressure on traditional licensing bodies to reform their laws and policies to allow some form of telehealth practice of medicine and other health care across state lines and borders within the European Union.

## Cost-Benefit Analysis of New Legislative Proposals

The US Congressional Budget Office (CBO) produces independent formal cost projections for every bill approved by Congress, including telehealth-related legislation. Although aided by a panel of 22 advisors from a variety of health care fields, the CBO’s current process of formulating cost estimates excludes many of the potential effects of health care and telehealth policy. None of the 21 cost estimate reports on telehealth legislation issued by the CBO in the last decade included an in-depth analysis of cost savings, efficiency, or qualitative impacts. By focusing largely on the short-term financial costs of new legislation, the CBO did not take into account potential cost savings, potential increases in the efficiency of health care resource use, or the value of quality improvements often associated with implementation of telehealth programs. Lack of cost-benefit analysis stymied past efforts to improve telehealth policies at the federal level and hindered the adoption and growth of telehealth programs across the nation. In contrast, European countries, such as the United Kingdom, Denmark, France, Germany, and Sweden, have long-standing Health Technology Assessment (HTA) organizations that advise government bodies on the costs and benefits of potential health technology treatments. These HTA organizations estimate the value of better clinical outcomes using scales such as quality-adjusted life years (QALYs), healthy years equivalent (HYE), and disability-adjusted life years (DALYs), among others, which weigh the potential qualitative effects of health care treatments and technologies against their potential financial costs.

## Telehealth Approaches for Cross-Sector Care Integration

In both the United States and the European Union, hospitals and public health care systems tend to be fragmented between hospitals and municipally based health care services. Among fragmented health care systems in the countries within the European Union and in individual states in the United States, the use of telehealth technologies can create jurisdictional conflicts, policy conflicts, and remain tangential to care practices rather than integrated into health care infrastructure. Collectively, barriers to integration may slow the development of a common vision for care, treatment, and rehabilitation of patients and minimize collaborative care among health care professionals across sectors [38].

Health care organizations in the United States, such as Kaiser Permanente or the Veterans Health Administration (VHA), have a single health care system: the hospital, district nursing, health care center, and primary care providers are integrated into a single organization. Such large systems also have a single, unified information technology system, an electronic health record (EHR) to coordinate and plan patients’ care processes, tend to have a high degree of adoption of telehealth solutions for patients with chronic diseases, and utilize a more innovative approach to testing new models of care based on patients’ preferences for using telehealth technologies within the health care system.

In both the United States and the European Union, telehealth technologies are being tested in many innovative ways to maximize emerging care models, including redesign of chronic disease management and the improvement of cross-sector care management. Examples of new models of care using telehealth technologies are home hospitalization of cardiac patients [39], preventive home monitoring of patients with COPD [40], and telerehabilitation of cardiac patients [41].

Over the last decade in the European Union, information technology solutions and telehealth technologies have been integrated between hospitals and municipalities and have reached a higher degree of data integration and sharing for the benefit of coordination and collaboration between health care professionals across sectors in patient care processes.

Examples of large EU telehealth projects with technologies involving both hospitals and municipalities are Renewing Health [42], United4Health [43], and MasterMind [44]. In the Renewing Health project, 8 countries participated (Denmark, Italy, Sweden, Norway, Spain, Finland, Greece, and Germany). The target group was patients with COPD, diabetes, and heart diseases, and a total of 7000 patients were enrolled in the study. Results from the project showed that the cross-sector telehealth solution led to a shift of tasks between health care professionals across sectors and there was an improvement in communication between professions and between sectors [42]. The health care professionals reported that patients took greater responsibility for their own health when they were able to see their own data. When patients could not see their data, the health care professionals felt that the patients were less responsible for their health care and that the two-way communication was limited [42].

## Emerging Issues That Influence Telehealth Delivery

Providing high-quality, accessible, and cost-effective health care remotely for a socially, economically, and financially diverse population presents challenges, whether within a country or between countries. The most substantial challenge is that of providing care for patients with chronic diseases. Fifty percent of patients in the United States have one or more chronic disease(s), accounting for 75% of the financial burden to the health care system [45]. In the European Union, 70% to 80% of health care budgets are spent on chronic diseases [46]. Establishing ways to lessen the burden and provide for these care needs requires systems that offer timely access to care, engage patients to participate, and prevent patients from inappropriate service utilization such as unnecessary emergency room visits. Telehealth is a viable alternative to standard face-to-face health care provider interactions.

A firm commitment to establishing large health care communication networks has existed for many years. As networks have grown, patient-provider communication and smaller handheld devices have been incorporated into the fabric of chronic disease management. Mobile phone apps and Web-based programs allow patients to manage their chronic diseases at home. It has been estimated that 93 million people in the United States have access to mobile phones, a number likely to increase in the future [47].

The telehealth technologies that are emerging are not only smaller and more efficient (eg, offering office-based desktop computer consultation with providers through patient-specific devices), but now include education for patients and may even offer suggestions for change in disease-specific treatment. Key issues driving the future of telehealth include (1) personalization of health care; (2) matching patients with appropriate technologies; (3) optimal use of health care data, including developing a secure interface between patient-generated data and the HER; (4) new education paradigms for patients and providers; (5) new communities of knowledge and practice; (6) new care and business models tailored to sustainability and scalability of telehealth initiatives; (7) transfer of scientific knowledge from research to implementation and practice; and (8) innovative research methodologies within telehealth. Each of these issues will be discussed subsequently.

## Personalization of Health Care

There is no “one-size-fits-all” approach to managing patient care with telehealth because chronic diseases management is diverse. For new technologies to succeed, they must accommodate a spectrum of user needs. Technology must engage patients in their care and enhance collaboration with the health care system or they are destined to fail. Patients need skills and tools to proactively apply vital technology information. In addition, patients need their use of new technology to be personally meaningful (ie, in terms of relevant self-care) because these devices can serve as an intrusion into the patient’s daily life and must serve to bring their health into focus at a personal level, not define them based on their disease state.

For example, patients with diabetes clearly do not use technology in a uniform manner. Patients with type 1 diabetes engage in accepting their disease and adapt to living with their disease by checking their blood glucose and monitoring what they eat [48,49]. These patients, who are usually younger and more adept at using handheld devices, mobile phone apps, and Web-based programs, tend to be earlier adopters of new technologies that help them log their caloric intake, follow finger-stick blood glucose samples, and monitor daily exercise routines. The key to successful technology-based treatment is getting these patients to participate in using monitoring systems and finding a way to provide ongoing feedback that keeps patients engaged. Providers must offer encouragement and meaningful insight into data throughout their progress or their continued participation may decline.

Patients with type 2 diabetes, who may be older and less familiar with technology, will likely apply technology in a different manner. Some have been dealing with their disease for many years, whereas others are confronted by a diabetes diagnosis only when in their fifties or sixties. Technology might not be as attractive to this group of patients compared to younger patients. Some patients with type 2 diabetes may have lost limbs, suffer from neuropathy, or have visual problems secondary to long-standing uncontrolled blood glucose. These populations, if they are to be successful, may require a different device and a different approach to using technologies. Access, familiarity with technology, ease of use, and size of text fonts are important. A range of devices should be available to meet the diverse needs of this group. Currently, telehealth systems store information from multiple sources: patient-collected physiological data, laboratory data, behavioral information, medication dosages, subjective symptoms of hypoglycemia, event data (eg, emergency room visits), and images (eg, retinal or wound photos) [50]. An all-encompassing approach might benefit patients who are extremely well organized and can handle a large amount of data, but some patients might be overwhelmed and would not participate in telehealth. Ultimately, there must be a match between technology, personalization, and the patients’ needs and wishes. Providers must match the proper device and data management approach to the proper patient. Health care providers need to be aware that some patients use telehealth as a means to get in contact with their providers and will also visit a nearby center for a face-to-face follow-up at the same time and for the same health issue.

### Matching Patients With Appropriate Technologies

As telemedicine and telecommunication have been lauded as a possible solution to the emerging shortage of health care providers, we need to remain cognizant that the use of technology in place of a face-to-face encounter will not be as easy for some patients as others. The US population is increasing and is estimated to grow by 20%—to 363 million—by the year 2030 [51]. The population is aging as well and those aged 65 years and older (12.4% of the population in 2000) will likely make up 19% of the US population by 2030. Accompanying this aging population is an increased incidence of chronic health conditions and expenditures associated with chronic disease management. Novel telehealth platforms require a match based on patient’s age, education, interests, physical capabilities, familiarity, access to technology, and support to help with self-care and functional independence. Computer-based desktop apps with large screens and static interaction may be best suited for an aged population of patients who have limited manual dexterity and visual limitations. For the elderly, the user interface often needs to be simple, easy to use, and provide meaningful interaction and feedback. Devices that allow patients to follow a script and alter care based on physiological information need to be efficient and user-friendly. Perceptual, motor, and cognitive abilities need to be considered when matching technology to patients.

A younger population that has been exposed to such technological advances would be more likely to use this type of monitoring device. The ability to follow one’s progress, compete with other patients in attaining preset goals, and receive immediate feedback would seem attractive to this younger group. Beyond establishing patient-device symbiosis, researchers have to weigh the intrusive nature of these devices and match patients who appreciate constant oversight with those who would prefer a more discrete means of monitoring. There is a distinct dichotomy between those patients who greatly enjoy having a daily reminder to take their pills, exercise, and eat right versus those who appreciate some early instruction and would then prefer to drop their monitoring altogether. Establishing patient preference in that arena will also take time and effort, and the original protocol for the patient might need to be altered. Either way, we must strive to provide patients with devices that maximize success by applying their strengths and avoiding reliance on functional weaknesses.

Matching patients with a proper device and gathering large amounts of meaningful data will lead to improved insight into a person’s disease state and better assessment of the success of care management strategies. The VHA serves as an example in that regard. The VHA established the first large-scale use of telehealth in 1977. In 2013, more than 600,000 veterans accessed VHA health care using a telehealth program. Established in 2003, the VHA Care Coordination/Home Telehealth (CCHT) network provides routine noninstitutional care and targets care management for patients with diabetes, chronic heart failure, hypertension, posttraumatic stress disorder, COPD, and depression. CCHT uses remote monitoring devices placed in the veteran’s home to communicate health status and to transmit biometric data that are monitored remotely by care providers [44]. At present, more than 70,000 patients concurrently participate in this program, which is projected to grow to reach more than 92,000 patients within the next few years. Analysis of ongoing data has allowed the VHA to change their approach and management strategies over the years, and serves to make the home the preferred place of care whenever possible and appropriate. Through use of telehealth, the VHA telehealth program reduced admissions by 20% in 2010 [52]. Patients with diabetes had a 20% decrease in resource utilization, those with heart failure had a 30% reduction, and those with depression had a 56% reduction [9]. Patient satisfaction remained above 86% and all but 10% of those approached were willing to participate in the program. Analysis of those patients who utilized the program suggests that the quality of care and patient-specific outcomes have not been compromised by utilizing the CCHT model. As the VHA program continues to grow, it is clear that it will be increasingly successful in gathering and analyzing telehealth data to better serve future patients with chronic diseases.

### Optimal Use of Health Care Data and Secure Interface Between Patient-Generated Data and the Electronic Health Record

Large health systems have much to gain by providing increased communication and patient engagement through mobile devices and Web-based interfaces. Beyond chronic disease management, secure methods of data capture working directly with patient communities open up major opportunities for wellness and health maintenance initiatives, as well as dynamic participation in research [53,54]. Yet there remain significant concerns regarding the ownership and obligations inherent in the communication of personal health data by health systems for data collected through patients’ mobile devices. Health systems are exploring fundamental issues such as when ownership of patient-provided data begins and what scope it encompasses. Given the need for third-party telecommunications carriers to support the connectivity of personal devices, and often independent developer apps to manage local capture of data, there remains a lack of clarity regarding the conditions under which personal data becomes protected data and the legal obligations this imposes on health policies such as the Health Insurance Portability and Accountability Act (HIPAA) [53]. Adding to this challenge are the many patients and health advocates who are frustrated by lack of accessibility to their own personal health information and associated overprotection of such information by privacy laws and paternalistic health institutions. This is a two-fold challenge: that of determining ownership and, ultimately, indemnity for data that can be collected by and from patients, while also deciding on strategies for data that are further aggregated and integrated with different sources within clinical systems. This dual challenge has influenced the lack of broader dissemination to date. Health care systems are inherently risk-averse. They struggle to keep up with the broad opportunities offered by these emerging technologies.

Two routes for obtaining patient-contributed data presently predominate: active participation, in which patients fill out and upload their own health information or test results, and passive participation, in which patients provide data through monitoring devices or other mechanisms that have limited interaction other than aggregate viewing (eg, personal fitness monitors). Both routes of collecting personal health data and the risk-averse policies of institutions are helping to clarify principles of data management. Health providers are moving toward support of a full and auditable “chain of custody” or data provenance support for patient communications in anticipation that health care communication derived from data may be called into question [55]. It should be noted that data provenance has a secondary benefit in that it improves the ability to define and address measures of quality and communications.

At an organizational level, both means of collecting personal health data are altering the roles of institutional data handlers, such as hospitals, clinicians, and testing companies, and are leading to changes in determining ownership of health data. Among proponents of personal ownership of health data, the removal of intermediaries is seen as a strength because it empowers individuals to control and deploy their information for chosen purposes. Among the critics of personal ownership of health data, however, there remain concerns that personal ownership will negatively impact the quality of data and have a subsequent impact on data quality used for the practice of health care.

Increasingly, patients are tracking their health status and incorporating lifestyle information into their overall health management. For the most part, this area of great innovation is taking place in the social media and has not yet been integrated into clinical care. Likewise, community-level data inform and shape health trajectories and health policies and are not well integrated into clinical care. Achieving individual and community improvements in health depends on building capacity to integrate data across sources into actionable packages so that individuals can act to improve their own health and communities can plan and deploy resources and policies to address barriers and facilitators to health attainment.

The incorporation of technology into health care settings, such as the adoption of EHRs, has grown substantially in the past few years, with nearly 40% of all physicians adopting basic EHR capabilities. Original policies pertaining to EHR technology created incentives and penalties that put an overwhelming emphasis on provider-centric health information technology (HIT) with EHR systems built to give providers better access to information and improved methods of storing and sharing that information between providers. The focus was placed on provider adoption, with minimal incentive for patients to engage and use the system. Notably absent in the legislation and goals of federal HIT is the voice of the patient in creating HIT that meets the needs of patients and can lead to meaningful health outcomes. The lack of focus on patients has come to the attention of numerous groups, including the American Telemedicine Association and Healthcare Information and Management Systems Society, which have joined five additional industry groups in advocating the inclusion of standards that require the EHR to incorporate patient-generated data from remote monitoring devices. Underlying this advocacy is the belief that the value of extending HIT requirements to include patient-generated data and data collected outside of traditional office visits will be realized through increased efficiency of delivery of health care services and systems that support timely exchange of data and information to improve health outcomes. Chronic disease registries and websites could accelerate progress in mobilizing appropriate evidence-based care in a timely fashion, promoting communication among the care team, and helping to design community or population-level interventions to improve health.

### Creating New Education Paradigms for Patients

Mobile phone and other emerging handheld applied telehealth-based instruments can be used as electronic (e)-learning tools for patient education, mobile clinical communication, and disease self-management education. When emerging telehealth tools and devices store a great amount of information, they can become the source tool for information sharing and education in examination rooms and at hospital bedsides, and electronically through e-transfer of information. Large wall- and desk-mounted screens are common in health care centers, and home televisions now have monitor functions. When telehealth tools are connected, the education experience may be enhanced.

Data from mobile phones and from internal or external telehealth instruments can be linked to desk-based or free-standing kiosk education devices or centers that will be prevalent in ambulatory centers of the future, and may even be tied into self-service electronic systems used to check-in to appointments, request medication refills, and provide other health care purposes. Patients with in-dwelling or externally applied cardiac (or other) devices that store data or allow for transfer of data to an external storage system will be able to access a kiosk-like education system, retrieve or synchronize data, receive education about the meaning of data, and receive instructions about the plan of care based on e-data findings. Plans for care, as part of the system, would have been previously vetted by health care professionals and be based on individualized algorithms to enhance safety and decrease the risk of harm. The innovative education roles of mobile phones and other technology continue to evolve with new software, hardware, innovative storage solutions, and patient confidentiality solutions.

There are numerous examples of e-learning tools in development and testing. In one report [48], researchers explored an application of Web 2.0 integrated with Internet-protocol television for personalized home-based health information in diabetes education among adults who were not strong computer and Internet users. This intervention provided diabetes educator-delivered, personalized health information directly to patients in their homes through an enhanced home television screen and remote control. The goal was to build health literacy and knowledge about diabetes management. After testing the system, researchers concluded that the system had educational potential [56]. In another study, parents of babies with infantile hemangiomas were trained to assess their children’s skin abnormalities for early complications through an e-learning module or by a dermatologist-delivered e-consultation [57]. After e-learning, parents’ judgments about diagnosis were found to be in concordance with those of the dermatologist in 96% of the cases. Results of this study indicate that correct diagnosis via e-learning can promote earlier recognition and treatment of infantile hemangioma risk factors and complications.

As new education paradigms emerge that use telehealth and other digital technology, it is important to recognize that there is a digital divide among patients. Those patients especially at risk of negative health outcomes could benefit most from telehealth tools. A qualitative analysis to gain better insight on the digital divide of patients demonstrated that patients’ gaps in knowledge of technology are greatest at three points: (1) in the clinical setting, when patients’ preexisting personal barriers to care are exacerbated; (2) during screening; and (3) during physician-patient follow-up [58]. Technology knowledge gaps can create confusion and fear, and patients may have low confidence in the quality of the content. Thus, when new education paradigms are created, overcoming the digital divide must be considered.

### Creating New Education Paradigms for Health Care Providers

Mobile phones and other emerging handheld devices are powerful and useful professional education tools for health care providers. Professional literature and educational materials can serve as conduits for information that enhance the practice of evidence-based medicine, provide professional education, act as a mobile clinical communication aid (with other health care professionals or office and hospital colleagues), store disease self-management education materials, and allow for remote (live or streamed) patient education. Apps are becoming more sophisticated and include static or motion images, such as in ophthalmological examinations [59], trauma in rural settings [60], and mobile phone and other telehealth tools served for multiple emerging purposes, including health care provider education. Adoption of high technology medical communication—in addition to high-performance computers, fiberoptic equipment, and high-resolution cameras—enables greater capacity for collaboration and learning between health care providers.

### Forming Communities of Knowledge and Practice

Given the significant changes in telehealth and telehealth applications brought about by the rapid emergence of health care organizational change, new software apps, new devices, and new forms of data, it is important for providers to form communities of expertise in applying the most recent scientific advances. App developers are rarely health care experts and patients may forget the “buyer beware” motto associated with software purchase or free downloading. One case in point is that of apps for pain, 80% of which are available for iPhones. Researchers found 220 apps, 80% of which were built on the iOS platform and ranged in price from free/nominal cost (generally less than US $5) to as much as US $90. Unfortunately, in 65% of the apps identified, there was no evidence of health care provider involvement in development, even though the primary purposes of these apps was pain education (24.1%), self-management of pain (62.3%), or both (13.6%) [61].

Further, not all telehealth systems successfully meet educational or behavioral outcomes. When telephone-based reminders and Web-based educational tools were used to improve medication adherence for acne treatment, only the Web-based educational tools had a positive effect [62]. When home video telehealth and monthly telephone counseling, respectively, were used to maintain weight in African-American women, there were no differences in outcomes for the two methods; however, valid digital video recorder use during the intervention period was reported as low, ranging from zero to 42% use per participant [63]. In an 18-month longitudinal study of telehealth for diabetes management, patients using telehealth required less support in physical activity, healthy eating, and problem-solving behaviors than control participants, but more support in medication adherence and healthy coping [64]. It might be that telehealth users became dependent on telehealth to promote medication adherence and provide coping advice. Although medication reminders and coping advice are beneficial, patients should become more independent in self-care over time and be able to overcome routine problems that come with managing diabetes. It will be increasingly important to conduct research on a range of telehealth education paradigms and tools to identify attributes of successful training paradigms and ensure that there are no issues related to the digital divide.

As telehealth becomes more prevalent, it will be important to ensure that communities of experts with knowledge of and experience with specific patient populations can develop systems and processes that ensure excellence in educational message content and can match the proper telehealth system with the intended education and clinical outcomes. It is important to use communities of experts to reassess the effectiveness of education content at frequent, regular intervals to ensure that best practice and evidence-based information are used, further ensuring that education (and self-management based on education) will promote optimal clinical and behavioral outcomes. Finally, as e-learning tools become more prevalent in private homes, there will be a greater need for communities of knowledge and practice related to privacy and information security. In a study of home rehabilitation programs for chronic pulmonary disease and diabetes, an assessment was made of e-diaries used to communicate with health care professionals, focusing on privacy, security, and risks to information security. Threats identified regarding data included those related to confidentiality, integrity, availability, and quality. From the perspective of a technical platform, the issue of confidentiality was identified as the most serious, in one case reaching an unacceptable “high-risk” level. Telehealth in the home offers additional threats to privacy and confidentiality compared to hospitals and health care centers that have controlled environments. Consequently, telehealth will require the development of commensurate levels of information security to support the rapid adoption of emerging telehealth tools.

## New Care and Business Models Tailored to Sustainability and Scalability of Telehealth Initiatives

In order to reduce risks and costs when starting up a new telehealth service, it is useful to develop new care and business models to increase the probability of success of the service.

To understand the dynamic and workflow of telehealth among health care professionals and within health care systems, a new theoretical framework for understanding cross-sector care integration needs to be developed. One way to develop such a framework is to employ an interorganizational approach, such as that used in the eHealth-enhanced Chronic Care Model [65]. A new framework should address specific approaches for cross-sector care integration, redesign of chronic disease care management, and redesign of multiple care practices through telehealth.

Most of today’s telehealth solutions are designed to provide monitoring functionality for a single chronic disease, even though most elderly adults have multiple chronic medical conditions. In the future, chronic disease management through telehealth technologies needs to be versatile in functionality and to be able to support patients with multiple diseases. Systems need to provide more options or become more patient-specific and personalized. Stratification tools are needed for matching patient preferences and health care providers’ recommendations to specific technology. Guidelines that assist patients in understanding how to use the technology, how the data are analyzed, and how to self-monitor their care need to be developed to help patients obtain a higher degree of self-management.

There exists no common conceptualization of business models and cases for telehealth in the literature [66]. However, it is important to break down the business model and business cases into components to understand and then construct sustainable and scalable telehealth initiatives. Sustainable business models must be developed to meet the demands of the many stakeholders in telehealth programs and to create value for a company as well as for the health care sector and patient. There is limited research on the use of business models and cases in telehealth. However, the identification of innovative telehealth business models is now of interest globally. The question is whether it is possible to develop a general model that can be used across countries and still be sensitive to different legal and operational structures of reimbursement and varied socioeconomic contexts between developed and developing countries. For example, the lack of transportation in developing countries will place a higher emphasis on critical access to basic health care, whereas the value proposition in a developed country would more likely emphasize convenience of location [64].

The self-tracking technologies are expected to change the role of the consumer in the future because we can expect that the consumer will be able to deal directly with the companies selling medical devices or devices for tracking pulse, sleep, etc.

### Innovative Research Methodologies Within Telehealth

Evaluation of the efficacy of telehealth has been carried out within the traditional research paradigm using randomized controlled trials (RCTs). RCTs are considered the highest level of evidence because of their high level of internal validity, but they are both expensive and time-consuming. With technologies being rapidly developed, the RCT evaluation paradigm may become too cumbersome and time-consuming for stakeholders and managers within the health care system, and policy makers often do not have time to await a scientific assessment of a given technology before they have to decide on budgets for the coming financial year. Alternatively, managers prefer evidence-based decision making and may request information about clinical impact, cost-effectiveness, patient perception, and organizational aspects of telehealth.

This has been demonstrated recently by Kidholm et al [67] in a study of European health care managers. Globally, there has been discussion about developing a new framework for assessing telehealth technologies at different levels of development. Within the European Union, a new multimodal telehealth assessment tool has been developed, called the Model for Assessment of Telemedicine Applications (MAST). MAST provides a multidisciplinary assessment of telehealth technologies [68]. The assessment process has three steps. First, the preliminary assessment in which the maturity of the technology and the organization using it must be assessed (eg, in the form of a feasibility study). Second, a multidisciplinary assessment of the outcomes of the telehealth application is conducted within seven domains: (1) health problem and characteristics of the application, (2) safety, (3) clinical effectiveness, (4) patient perspectives, (5) economic aspects, (6) organizational aspects, and (7) sociocultural, ethical, and legal aspects. Finally, the third assessment step is a transferability assessment, in which the transferability of the evidence to the local setting is considered. The MAST model is currently the most widely used framework for evaluating telemedicine in the European Union and is used in a number of EU projects. Examples of MAST are available in studies by Minet et al [69] and Rasmussen et al [70].

### Transfer of Scientific Knowledge: From Research to Implementation and Practice

Telehealth offers the opportunity to deliver care that is accessible, convenient, and patient-centered, overcoming many of the barriers inherent in traditional health care delivery systems [71]. However, widespread implementation will require attention to systems engineering approaches to health care design so that it can address incentives, technical and human requirements, work processes, and payment issues [72]. To demonstrate and realize added value to health outcomes, telehealth implementation is not simply a feature to be added to existing health care delivery. It must be integrated into innovation at the system level. Integration involves examining the current flow of care for targeted subpopulations and revising the overall approach to care, integrating telehealth, and changing traditional elements. For example, using telehealth to manage chronic disease might incorporate interprofessional involvement, with nurses, pharmacists, or dietitians coaching the patient through telehealth between visits for primary care. Integration may require challenging adjustments in the current delivery of care. For example, the number of planned primary care visits may be reduced as telehealth is used to augment care.

For telehealth to be fully integrated into global health systems, a number of items that support system transformation will be needed. Given that telehealth often includes patient-generated data, significant changes will be needed to insure accurate, efficient, and timely monitoring of health parameters that are useful for guiding clinical decision making. Integration and interpretation of these data are essential to optimizing telehealth, yet many EHR systems do not have the capacity to incorporate patient-generated data nor are they not able to make it available in a time-sensitive fashion. Similarly, new competencies will be required for health care professionals in telehealth and systems engineering to improve health [72]. Finally, telehealth research needs to promote approaches to care that are amenable for adoption in practice. The age-old challenge is to translate research findings into practice to facilitate adoption of new knowledge to telehealth. The challenges are to reinforce the urgency with which evidence is needed to drive policy and provide greater incentive for researchers and practitioners to collaborate.

### American and European Visions for Personalized Telehealth

In the United States, the Health Resources and Services Administration works to increase and improve the use of telehealth to meet the needs of underserved people, including those living in rural and remote areas, with low income, uninsured, or those enrolled in Medicaid. The Affordable Care Act is driving changes in health care delivery that bring greater value and access, particularly to populations who require complex care. As reimbursement moves from fee-for-service to value-based and outcome-driven payment, incentives for providing telehealth should improve. Through the Federal Office of Rural Health Policy and the Office for the Advancement of Telehealth (OAT), resources are provided in the United States to support regional telehealth technical assistance centers, a national telehealth policy center, and a national telehealth technology assistance center. In addition, OAT provides grants for the creation of evidence-based tele-emergency networks and for demonstration projects to test the use of telehealth networks in improving health care services for medically underserved populations in urban, rural, and frontier areas of the country.

The World Health Organization (WHO) has developed a European policy framework and strategy for the 21st century called “Health 2020.” The vision of this initiative is to achieve the highest level of health among European countries and to improve health for all citizens, and reduce health inequalities, empower citizens to take care of their own health, and strengthen people-centered health system and public health capacity [73]. In 2011, WHO launched a policy on health technology assessment of medical devices with a focus on this area because new technologies are evolving rapidly [74].

Similarly, the EU Commission launched an eHealth Action Plan for 2012-2020 entitled “Innovative Healthcare for the 21st Century in the EU” [75]. This plan seeks to improve chronic conditions, multiple morbidity management, and strengthen prevention; increase patient-/citizen-centric care via citizen empowerment and organizational sustainability; stimulate cross-border health care, security, and equity; and improve legal and market conditions for developing eHealth products and services [75].

Finally, Denmark has a national strategy for digitalization of the Danish public sector by 2020.0 [76]. Focus is on implementing telehealth at scale, improving personalized telehealth, quality of life for patients and citizens within the health care and social sectors, and to increase the efficiency and effectiveness of workflows within the public sector.

Within the European Union, the “Horizon 2020” research program offers funding possibilities to facilitate more telehealth projects at scale, with an implementation focus, and for international (US and EU) partners. The TTRN advocates more transatlantic telehealth studies to develop synergy in research and gain generalizable results at a more rapid pace.

## Personalized Telehealth in the Future: A Global Research Agenda

As telehealth plays an even greater role in global health care delivery, it will be increasingly important to develop a strong evidence base of successful, innovative telehealth solutions that lead to scalable and sustainable telehealth programs. A broad, multinational research agenda can provide a uniform framework for identifying and rapidly replicating best practices, while concurrently fostering global collaboration in the development and rigorous testing of new and emerging telehealth technologies. As an initial effort toward a global research agenda, the members of the TTRN offer a 12-point research agenda that incorporates health care parameters across mediated and traditional modes of care for the benefit of providers, companies, policy makers, and the international research community (see Textbox 1).

**Focus areas for personalized telehealth research.**

1. *Patient*

- Assessment of personal engagement in own health through the use of telehealth technologies (quantified self)
- Self-determination and motivation with regard to the use of new telehealth technologies
- Health literacy, eHealth literacy, technology literacy, contributions to design features of technology, and interaction with telehealth technologies
- Patient-to-patient interventions

2. *Home*

- Integration of smart home telehealth technologies (wellness and health devices and software, Internet of Things)

3. *Health care professionals*

- Communication for and between providers and patients (telehealth through mobile, wearable, and remote monitoring)
- Telehealth training and education, including designing communities of knowledge and practice

4. *Health system design, organization, and practice*

- Cross-sector integration using telehealth technologies (Accountable Care Organizations, bundled care, medical homes)
- Telehealth in redesign of chronic disease management
- Adoption of telehealth programs in clinical practice

5. *Technologies*

- Use of self-tracking technologies
- Design of user-friendly technologies
- Development of sensor technologies for detection of fluid in the body, sleep patterns, etc

6. *Data systems and infrastructure*

- Integration of telehealth devices with electronic health records and cloud databases
- Integration of personal health records data and telehealth devices and systems

7. *Data analytics*

- Algorithms for multimodel data platforms, devices, and sources
- Innovative data analytic approaches for integrating data for precision medicine, including predictive, personalized, and customized analytics

8.*Development of new telehealth technologies*

- Assessing mobile, intelligent, and individualized telehealth technologies
- Enhancing the matching of patient preferences and telehealth use
- Anticipation of telehealth innovations still to be invented
- International telehealth technology standards

9.*Research methods*

- Multidisciplinary assessment of the effectiveness of new telehealth services
- Advances in tracking, data transmission, and storage of telehealth data (real-time analytics vs store-and-forward)
- Rapid cycle design evaluation vs traditional randomized controlled trials

10. *Financing*

- Assessing innovative payment and reimbursement systems, especially in the emerging value-based health care environment
- Global variations in financing and paying for telehealth

11.*Privacy and security policy*

- Addressing different cultures of privacy (ethical issues) for patients
- Enhancing telehealth data security (given advances in mobile, wearable, and cloud-based system configurations)
- Local, regional, and international regulatory requirements (licensing, guidelines, standards)

12.*Public policy*

- Telehealth across state and international borders
- Professional licensing and standards
- Variation in intergovernmental and international telehealth policies and financing

The research goals are designed to facilitate comparative evaluations of telehealth solutions at multiple levels, from individual to system level, using a variety of devices and technologies, and in multiple settings and contexts. Although this research agenda requires specific refinements to address country and health system variations, it can provide a comprehensive orientation for pursuing global research in personalized telehealth.

## Abbreviations

CBO: Congressional Budget Office
CCHP: Center for Connected Health Policy
CCHT: Care Coordination/Home Telehealth
COPD: chronic obstructive pulmonary disease
DALY: disability-adjusted life years
EHR: electronic health record
HIT: health information technology
HTA: Health Technology Assessment
HYE: healthy years equivalent
IT: information technology
MAST: Model for Assessment of Telemedicine
OAT: Office for the Advancement of Telehealth
QALY: quality-adjusted life years
RCT: randomized controlled trials
RPM: remote patient monitoring
TTRN: Transatlantic Telehealth Research Network
VHA: Veterans Health Administration
WHO: World Health Organization
WSD: Whole System Demonstrator
